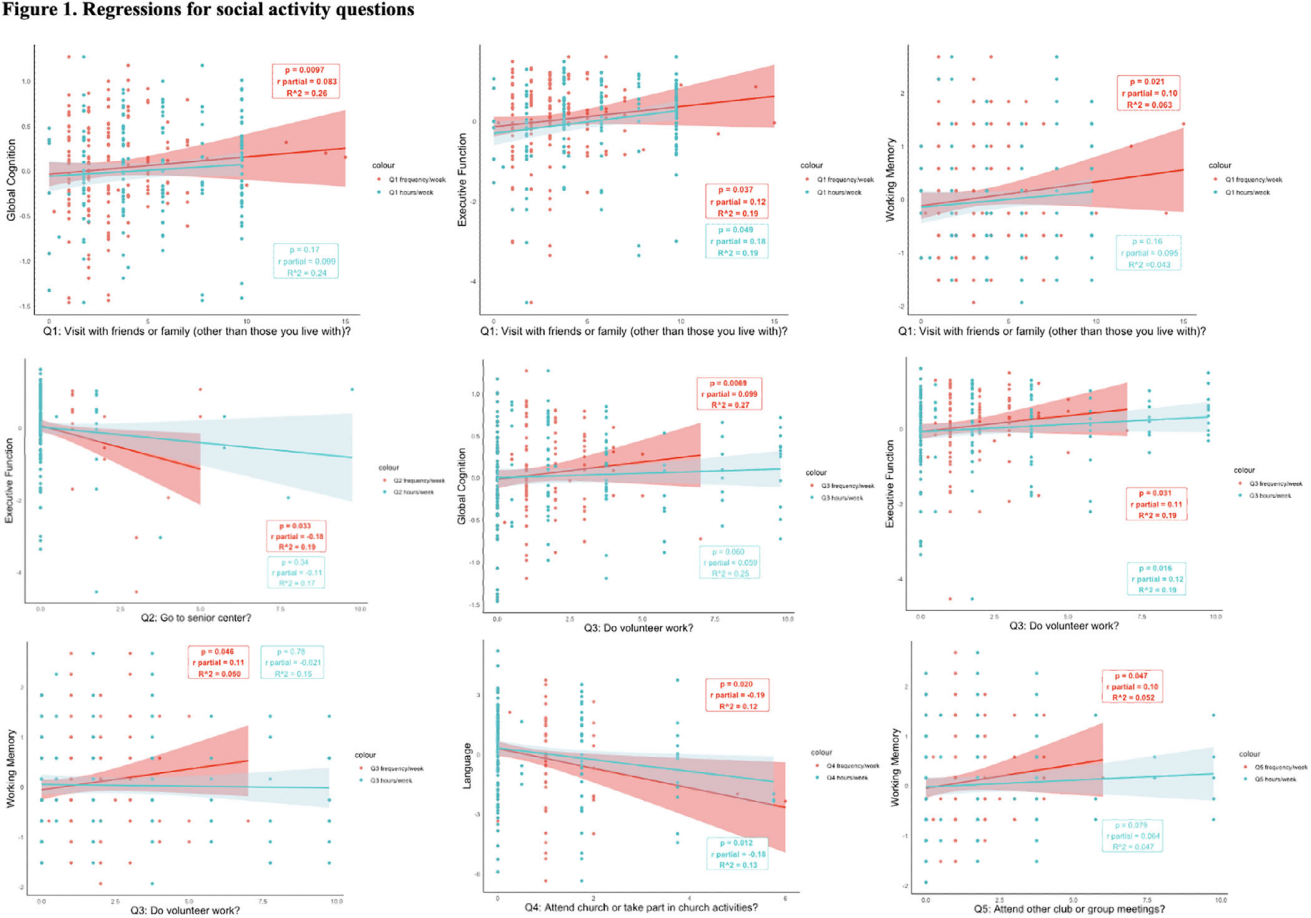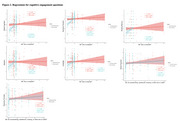# Association of types of cognitive and social activities with cognitive performance and Alzheimer's disease biofluid biomarkers: an examination of cognitively normal adults in the Stanford Aging and Memory Study (SAMS)

**DOI:** 10.1002/alz70860_107011

**Published:** 2025-12-23

**Authors:** Olivia Lu, Alexandra N. Trelle, Christina B. Young, Elizabeth C. Mormino, Anthony D. Wagner, Zihuai He, Valerie A. Carr, Sharon J Sha, Hillary Vossler, America Romero, Jennifer Park, Irina Anna Skylar‐Scott

**Affiliations:** ^1^ Stanford University, Palo Alto, CA, USA; ^2^ Stanford University School of Medicine, Stanford, CA, USA; ^3^ Department of Neurology and Neurological Sciences, Stanford University School of Medicine, Stanford, CA, USA; ^4^ Wu Tsai Neuroscience Institute, Stanford, CA, USA; ^5^ San Jose State University, San Jose, CA, USA; ^6^ Wu Tsai Neurosciences Institute, Stanford University, Stanford, CA, USA

## Abstract

**Background:**

Non‐pharmacological approaches may guide prevention and treatment strategies for patients with Alzheimer's disease (AD), but little is known about the “dose” and “type” required. The goal of this study is to examine what types of social and cognitive activities are associated with cognitive performance and AD biomarkers.

**Method:**

173 cognitively normal participants (69.0 ± 6.4 years) completed a questionnaire regarding frequency and hours per week of 7 social and 5 cognitively engaging activities. Participants underwent neuropsychological testing (z‐scored composites of executive function, working memory, attention, episodic memory, visuospatial function, and language as well as a global cognitive composite), CSF biomarker testing (127 individuals: Aβ‐42/Aβ‐40, *p*‐tau181, and t‐tau), and plasma testing (127: Aβ‐42/Aβ‐40 and pTau181). Regression co‐variates included age, sex, education, and APOE. Because this was a hypothesis‐driven analysis, multiple comparisons corrections were deferred.

**Result:**

Visiting loved ones more frequently was associated with higher global cognition (β=0.20, *p* = 0.0097), executive function (β=0.17, *p* = 0.037), and working memory (β=0.20, *p* = 0.021) scores. More hours spent on these visits was associated with higher executive function scores (β =0.15, *p* = 0.049). Volunteering more frequently was positively associated with global cognition (β=0.20, *p* = 0.0069), executive (β=0.17, *p* = 0.031), and working memory (β=017, *p* = 0.046); volunteering for more hours per week was positively associated with executive function (β=0.18, *p* = 0.016). There was a negative association between cognitive performance and senior center attendance (executive function: β=‐0.17, *p* = 0.033) and church attendance (language and frequency: β=‐0.21, *p* = 0.012; language and hours: β=‐0.19, *p* = 0.020). Attending clubs more frequently was positively associated with working memory (β=0.17, *p* = 0.047). Using computers for longer was significantly associated with global cognition (β=0.26, *p* <0.001), executive function (β=0.29, *p* <0.001), working memory (β=0.17, *p* = 0.045), attention (β=0.18, *p* = 0.026), and language (β=0.18, *p* = 0.029). Additionally, doing woodworking, needlework, drawing, or other crafts for longer was positively associated with executive function (β=0.15, *p* = 0.048). Games, billiards, attending events, playing an instrument, and reading were not associated with cognitive performance. There was no association between these activities and AD biomarkers.

**Conclusion:**

In a comprehensive analysis of the associations between types of social and cognitive activities on cognitive scores, social visits, volunteering, clubs, computer use, and crafts were associated with higher cognitive performance.